# A proof-of-concept study to evaluate the efficacy and safety of BTI320 on post-prandial hyperglycaemia in Chinese subjects with pre-diabetes

**DOI:** 10.1186/s12902-018-0288-5

**Published:** 2018-08-31

**Authors:** Andrea O. Y. Luk, Benny C. Y. Zee, Marc Chong, Risa Ozaki, Carl W. Rausch, Michael H. M. Chan, Ronald C. W. Ma, Alice P. S. Kong, Francis C. C. Chow, Juliana C. N. Chan

**Affiliations:** 1 Department of Medicine and Therapeutics, The Chinese University of Hong Kong, Shatin, Hong Kong; 2 Li Ka Shing Institute of Health Science, The Chinese University of Hong Kong, Prince of Wales Hospital, Shatin, Hong Kong; 3 School of Public Health and Primary Care, The Chinese University of Hong Kong, Prince of wales Hospital, Shatin, Hong Kong; 4 Boston Therapeutics Inc., 354 Merrimack Street #4, Lawrence, MA 01843 USA; 5 Department of Chemical Pathology, The Chinese University of Hong Kong, Prince of Wales Hospital, Shatin, Hong Kong; 6 Diabetes and Endocrine Research Centre, The Prince of Wales Hospital, Shatin, New Territories Hong Kong

**Keywords:** Galactomannans, Prediabetes, Fructosamine

## Abstract

**Background:**

Galactomannan(s) are plant-derived fiber shown to reduce post-prandial blood glucose by delaying intestinal absorption of carbohydrates and slowing down gastric emptying. We examined glucose-lowering effects of BTI320, a propriety fractionated mannan(s) administered as a chewable tablet before meal in a proof-of-concept study in Chinese subjects with prediabetes.

**Methods:**

Sixty Chinese adults aged 18–70 years with either impaired fasting glucose, impaired glucose tolerance, or glycated haemoglobin 5.7–6.4% (39-46 mmol/mol), were randomly assigned in 2:2:1 ratio to either BTI320 8 g (high dose), BTI320 4 g (low dose) or matching-placebo three times daily before meal for 16 weeks. The primary endpoint was change in fructosamine in subjects treated with BTI320 compared with placebo from baseline to week 4. Indices of glycaemic variability based on continuous glucose monitoring (CGM) and standard meal tolerance test were explored in secondary analyses.

**Results:**

Of 60 subjects randomized, 3 subjects discontinued study treatment prematurely. In intention-to-treat analysis, no significant differences in change in serum fructosamine between low or high dose BTI320 and placebo were observed. Using random effect models, adjusted for variability by meals, treatment with low dose BTI320 was associated with reduction in 1-h (*p* < 0.01), 2-h (*p* = 0.01) and 3-h (*p* = 0.02) post-prandial incremental glucose area-under-curve and post-meal maximum glucose (*p* = 0.03) compared with placebo. Subjects receiving low dose BTI320 had greater body weight reduction than placebo group.

**Conclusions:**

BTI320 did not change fructosamine levels compared with placebo. BTI320 reduced glycaemic variability based on CGM indices.

**Trial registration:**

The study was registered at www.clinicaltrials.gov, reference number NCT02358668 (9 February 2015).

**Electronic supplementary material:**

The online version of this article (10.1186/s12902-018-0288-5) contains supplementary material, which is available to authorized users.

## Background

One in ten Chinese adults have diabetes and recent estimates from the International Diabetes Federation indicates that there are 11 million people living with diabetes in China [1, 2]. Individuals with impaired glucose tolerance (IGT) or impaired fasting glucose (IFG) are at increased risks of developing diabetes at estimated annual conversion rates of 3–10% depending on the presence of other risk factors [3]. Diabetes may be prevented or delayed through intensive lifestyle intervention and pharmacological treatment agents [4–7]. The Diabetes Prevention Program demonstrated that lifestyle modification reduced progression to diabetes by 58% and metformin by 31% in people with pre-diabetes during the 2.8-year in-trial period, and that the benefits persisted at up to 15 years post-intervention albeit attenuated [4, 8]. Similarly, the 3.3-year STOP-NIDDM trial reported 25% relative risk reduction of incident diabetes with acarbose compared to placebo in people with IGT [7]. Despite the best clinical evidence and international guidelines, the effects of diabetes prevention programs are often limited and not sustained in real world setting due to poor uptake and persistence as well as the safety concern of systemic drug product exposure. As such, the rising burden of type 2 diabetes and its associated morbidity and mortality remain a global health problem of enormous proportion [2].

A simple, non-systemic pharmacological approach to disease management is a universal healthcare ideal, and extracts of natural materials represent an explored opportunity. Specifically, galactomannan(s) are the active ingredient in natural gum and are used extensively in food industry as a thickener of free water [9]. Through its action in increasing viscosity of gastrointestinal content, carbohydrates are slow to interact with digestive enzymes, glucose absorption is delayed, and this results in diminution of post-prandial blood glucose excursion [10]. Galactomannan(s) have been previously examined in humans for its beneficial effects on blood glucose, blood cholesterol and body weight, although most of these studies were of small sample sizes with notable heterogeneity in doses and preparation of the plant-derived gum tested [11–17].

BTI320 is a proprietary combination of fractionated mannans derived from guar gum and other plant sources and is administered in the form of a chewable tablet. In an earlier open-label study of 24 patients with type 2 diabetes, BTI320 8 g and 16 g taken before a test meal reduced 3-h post-prandial glucose area-under-curve (AUC) in 75% of patients [18]. The main adverse events reported in that study were increased flatulence and bloating. Here, we examined the glycaemic efficacy, tolerability and safety of 16 weeks’ intervention with BTI320 compared with placebo in Chinese adults with prediabetes. In the present proof-of-concept study, we utilized a continuous glucose monitor (CGM) device to monitor glucose levels at 3 multi-day periods throughout the 16 weeks’ study to explore the effects of BTI320 on post-prandial glucose excursion and variability.

## Methods

### Study design and subjects

We undertook a randomized, double-blind, placebo-controlled, parallel arm study with the first subject enrolled on 30 March 2015 and the last subject completed the study on 19 February 2016. The study was conducted in the Diabetes and Endocrine Research Centre of the Chinese University of Hong Kong (CUHK) at the Prince of Wales Hospital, Hong Kong Special Administrative Region. Subjects were identified from non-specialist general medical or family medicine clinics at the hospital. We recruited Chinese subjects aged between 18 and 70 years inclusive, fulfilling at least two of the following three criteria: 1) fasting plasma glucose 5.6–5.9 mmol/L (IFG) and/or 2-h plasma glucose 7.8–11.0 mmol/L (IGT) during a standard 75 g oral glucose tolerance test (OGTT); 2) glycated haemoglobin (HbA1c) 5.7–6.4% (39–46 mmol/mol); and 3) at least one of the following risk factors of a) history of gestational diabetes, b) history of diabetes in first degree relatives, and c) two or more of metabolic syndrome components of triglyceride ≥1.7 mmol/L, high density-lipoprotein (HDL) cholesterol < 1.3 mmol/L in women or < 1.1 mmol/L in men, waist circumference ≥ 80 cm in women or ≥ 90 cm in men, or blood pressure (BP) ≥130/80 mmHg. Exclusion criteria included current use of dietary supplements known to affect glucose or galactose metabolism, use of anti-diabetic medications in the previous 6 weeks, cardiovascular disease in the recent 12 months, renal impairment with estimated glomerular filtration rate < 60 mL/min/1.73m^2^, history of eating disorder, and known lactose or galactose intolerance. The study was registered at www.clinicaltrials.gov, reference number NCT02358668.

### Randomisation

The randomization process involved the use of computer-generated random numbers. Treatment group assignment of each sequentially randomised subject were contained in individually sealed, opaque and consecutively numbered envelops, which were opened by a non-study personnel.

### Intervention

Subjects meeting eligibility criteria were randomly assigned to receive BTI320 4 g (*n* = 24), BTI320 8 g (*n* = 24) or matching placebo (*n* = 12) orally three times daily, 10 min before each main meal for 16 weeks. Each 4-g tablet of BTI320, administered as a chewable tablet, contained 2.0 g of the key ingredient mannan polysaccharides. Other ingredients included food grade sorbitol, magnesium stearate, malic acid, natural flavors and colors. Subjects were instructed to maintain their usual dietary pattern and physical exercise levels. Subjects were reviewed every 4 weeks for assessment of adverse events and drug compliance, the latter was established by counting the returned tablets.

### Clinical measurements

Serum fructosamine was measured at baseline and 4-weekly interval until completion of treatment at 16 weeks, HbA1c at baseline and 16 weeks, and OGTT at screening and 30-day post-treatment visit. Meal tolerance test (MTT) using a standardized meal of 500 kcal was conducted at baseline, 4 weeks and 16 weeks measuring plasma glucose, insulin, C-peptide and glucagon-like peptide (GLP)-1 at 0, 15, 30, 60, 90 and 120 min. The standard meal consisted of two pieces of pineapple shortcakes and one carton of soymilk with nutritional breakdown as follows: carbohydrates 75.7 g (57.1% of total energy intake), fat 21.2 g (35.9% of total energy intake) and protein 9.3 g (7.0% of total energy intake). Seventy-two-hour CGM using the Medtronic iPro®2 CGM and Enlite sensor was performed at baseline, 4 weeks and 16 weeks. Other metabolic parameters (body weight, waist circumference, BP, lipid [total cholesterol, HDL-cholesterol, triglyceride, low density-lipoprotein cholesterol], high-sensitivity C-reactive protein [hs-CRP]) and safety parameters (renal function, liver function and complete blood count) were measured at regular intervals.

Fructosamine was measured using colorimetric test by reaction with nitroblue tetrazolium. The measuring range of the fructosamine assay was 14–1000 μmol/L, intra-assay coefficient of variations (CVs) were 0.8% and 0.5% at concentration of 275 μmol/L and 515 μmol/L, respectively, and inter-assay CVs were 1.5% and 1.2% at concentrations of 262 μmol/L and 489 μmol/L, respectively. Glycated haemoglobin was measured using immunoassay traceable to the National Glycohaemoglobin Standardisation Program and the International Federation of Clinical Chemistry standards. The measuring range of HbA1c assay was 0.3–3.4 g/dL, inter-assay CVs were 1.2% and 0.7% at concentrations of 5.3% Hb and 9.6% Hb, respectively, and inter-assay CVs were 2.2% and 1.9% at concentrations of 5.0% and 10.4% Hb, respectively. Insulin was measured by immunoassay which had a measuring range of 2–300 mIU/L with intra-assay CVs of 3.6% and 2.9% at concentrations of 11.7 mIU/L and 51.2 mIU/L, respectively, and inter-assay CVs of 6.7% and 5.3% at concentrations of 11.2 mIU/L and 47.4 mIU/L, respectively. C-peptide was measured using immunoassay which had measuring range of 0.1–20 μg/L with intra-assay CV of 2.8% and 1.7% at concentrations of 0.7 μg/L and 6.2 μg/L, respectively, and inter-assay CVs of 3.5% and 6.3% at concentrations of 0.8 μg/L and 6.3 μg/L, respectively. Glucagon-like peptide 1 was measured by enzyme-linked immunosorbent assay (Immuno-Biological Laboratories Co. Ltd., Japan). The measuring range of GLP-1 was 1.25–80 pmol/L, intra-assay CVs were 9.8% and 2.2% at concentrations of 5.0 pmol/L and 7.8 pmol/L, respectively, and inter-assay CVs were 10.3% and 5.7% at concentrations of 6.1 pmol/L and 11.0 pmol/L, respectively. Glucose, total cholesterol, HDL-cholesterol and triglyceride were measured using the enzymatic colorimetric method. Insulin and C-peptide were analysed by the Siemens IMMULITE® 2000 XPi Immunoassay System, HbA1c was measured on the Roche Cobas Integra 800 System (Roche Diagnostic GmbH, Mannheim, Germany), GLP-1 was measured manually, and the rest of the assays were measured on the Roche Cobas c8000 Analytical System (Roche Diagnostic GmbH, Mannheim, Germany). All laboratory tests were performed in the Department of Chemical Pathology, the CUHK, the Prince of Wales Hospital, which was accredited by the National Association of Testing Authorities, Australia and the Royal College of Pathologists of Australasia for medical testing.

All subjects completed Food Frequency Questionnaire, Hill and Blundell questionnaire on appetite, International Physical Activity Questionnaire, and World Health Organisation Quality of Life questionnaire at baseline, 4 weeks and 16 weeks.

### Efficacy endpoints

The primary endpoint was change in serum fructosamine in subjects treated with low dose and high dose BTI320 compared with placebo from baseline to 4 weeks. The main secondary endpoints were changes in calculated indices of glycaemic variability (mean post-prandial incremental AUC [AUCpp] at 1 h, 2 h and 3 h, mean post-meal maximum glucose [MPMG], AUC-180, mean amplitude of glucose excursion [MAGE], standard deviation [SD], and percent CV) based on CGM data in subjects treated with low dose and high dose BTI320 compared with placebo during the study. The AUCpp is the area above pre-prandial glucose starting from the beginning of each main meal to 1 h, 2 h and 3 h after the meal, obtained using the trapezoidal rule. The MPMG is the mean maximal glucose value within 3 h after each main meal. The AUC-180 is the AUC for glucose level above 180 mg/dL (10 mmol/L). The MAGE is the mean difference in glucose values between consecutive peaks and nadirs, only considering changes above and below mean glucose of more than 1 SD [19]. The percent CV is SD divided by mean glucose values. Other secondary endpoints included changes in HbA1c, 2-h AUC of plasma glucose, insulin, C-peptide and GLP-1 post-MTT, body weight, BPs, lipids, hs-CRP, as well as changes in self-reported dietary intake and satiety from baseline to end of treatment in subjects treated with low dose and high dose BTI320 compared with placebo.

### Safety endpoints

Laboratory safety variables analyzed were renal function, liver function and complete blood counts. Self-reported adverse events including hypoglycaemic events were captured and analyzed.

### Statistical analysis

In our estimation of sample size, we assumed a mean serum fructosamine level of 273 μmol/L with SD of 22.5 μmol/L in the placebo arm, and a change of 10% in fructosamine level would be detected using a two-sided 5% level test with 80% power if there were 11 subjects per arm.

Efficacy analyses were performed in the intention-to-treat population which consisted of all randomized subjects who have received at least one dose of the assigned treatment. A *per protocol* analysis was also performed in subjects who have taken at least 70% of the treatment. Analysis of covariance (ANCOVA) was used to measure the changes in serum fructosamine, and changes in other glycaemic and metabolic indices from baseline to week 4 and week 16 between intervention arms, adjusted for age, gender and baseline measurements. The effects of low or high dose BTI320 compared with placebo on CGM glycaemic variability indices were further explored using random effect models with repeated measurements adjusted for intra-individual between-meal and between meal-day variability, age and gender. Linear mixed effect is a common statistical method to address repeated measurements [20, 21]. Post-hoc subgroup analysis was conducted on significant CGM glycaemic variability indices by dividing the population into 1) Low and high body mass index (BMI) stratified by the population BMI median; 2) Younger and older age groups by population age median; and 3) With IFG and/or IGT at baseline and without IGF and IGT at baseline. Analysis was performed using Statistical Analysis Software Version 9.4.

## Results

### Subject disposition and baseline clinical characteristics

A total of 77 subjects were screened and 60 subjects met eligibility for randomisation (Additional file 1: Figure S1). Twenty-four subjects were assigned to treatment with low dose BTI320, 24 subjects to high dose BTI320, and 12 subjects to placebo. Two subjects receiving low dose BTI320 withdrew from the study due to adverse events (one withdrew due to serious adverse event of osteosarcoma, and another due to abdominal pain), and 1 subject receiving high dose BTI320 withdrew consent for non-medical reasons. Overall 55 subjects have taken more than 70% of the study treatment and were included in the *per protocol* analysis.

The mean age of the cohort was 56.4 ± 9.1 years and 46.7% were male. At baseline, 4 subjects (6.7%) had IFG only, 23 subjects (38.3%) had IGT only, 15 (25.0%) had both IFG and IGT, and 18 (30.0%) had normal fasting glucose and glucose tolerance but HbA1c between 5.7–6.4% (39–46 mmol/mol). Mean serum fructosamine and HbA1c were 272.1 ± 19.9 μmol/L and 6.0 ± 0.3% (42 ± 2.1 mmol/mol), respectively. Glycaemic indices were comparable among the three intervention arms at baseline (Table 1).

**Table 1:** Baseline clinical characteristics of subjects in low dose BTI320, high dose BTI320 and placebo groups

	Placebo	Low Dose BTI320	High Dose BTI320
Number	12	24	24
Demographics			
Age, years	57.1 ± 10.9	54.1 ± 8.6	58.5 ± 8.5
Male, % (*n*)	25.0 (3)	54.2 (13)	50.0 (12)
Metabolic Parameters			
Body weight, kg	63.9 ± 20.0	74.2 ± 16.9	71.0 ± 16.2
Body mass index, kg/m^2^	25.1 ± 4.3	28.0 ± 5.8	26.9 ± 4.4
Waist, cm	88.0 ± 15.7	95.0 ± 15.6	90.6 ± 9.1
Systolic BP, mmHg	127.8 ± 8.7	121.7 ± 13.2	125.4 ± 16.2
Diastolic BP, mmHg	80.4 ± 7.3	78.4 ± 6.8	78.7 ± 7.2
Total cholesterol, mmol/L	5.3 ± 0.8	4.9 ± 1.1	4.9 ± 1.0
LDL-cholesterol, mmol/L	3.3 ± 0.6	2.9 ± 0.9	2.9 ± 0.8
Triglyceride, mmol/L	1.4 ± 0.6	1.2 ± 0.4	1.4 ± 0.8
HDL-cholesterol, mmol/L	1.5 ± 0.3	1.5 ± 0.3	1.4 ± 0.4
Fructosamine, μmol/L	278.9 ± 22.0	268.5 ± 18.3	272.2 ± 20.2
HbA1c, % (mmol/mol)	6.1 ± 0.3 (43 ± 2.2)	6.0 ± 0.3 (42 ± 2.1)	6.0 ± 0.30 (42 ± 2.1)
Hypertension, % (*n*)	58.3 (7)	45.8 (11)	54.2 (13)
Dyslipidemia, % (*n*)	33.3 (4)	25.0 (6)	41.7 (10)
Obesity, % (*n*)	25.0 (3)	16.7 (4)	20.8 (5)
Glycemic Status			
IFG, % (*n*)	0.0 (0)	12.5 (3)	4.2 (1)
IGT, % (*n*)	41.7 (5)	41.7 (10)	33.3 (8)
Both IFG/IGT, % (*n*)	33.3 (4)	16.7 (4)	29.2 (7)
NGT and HbA1c 5.7–6.4% (39–46 mmol/mol) only, % (*n*)	25.0 (3)	29.2 (7)	33.3 (8)
CGM Parameters			
1-h AUCpp, mmol/L×hour	6.33 ± 0.64	5.91 ± 0.53	6.22 ± 0.72
2-h AUCpp, mmol/L×hour	13.49 ± 1.43	12.68 ± 1.21	13.64 ± 1.87
3-h AUCpp, mmol/L×hour	20.20 ± 2.20	18.90 ± 1.69	20.20 ± 2.59
72-h AUC-180, mmol/L×hour	2.73 ± 7.64	0.40 ± 1.85	1.71 ± 3.35
MBG, mmol/L	6.45 ± 0.54	6.01 ± 0.45	6.20 ± 0.63
MPMG, mmol/L	8.07 ± 1.00	7.45 ± 0.78	8.20 ± 1.31
MAGE, mmol/L	3.21 ± 3.16	2.06 ± 0.69	2.68 ± 1.01
CV, %	18.00 ± 7.60	15.49 ± 4.43	19.07 ± 6.61
SD, mmol/L	1.18 ± 0.59	0.93 ± 0.28	1.19 ± 0.44

Expressed as mean ± standard deviation, or percentage (number) as appropriate

*AUC* area-under-curve, *AUCpp* post-prandial incremental area-under-curve, *BP* blood pressure, *CGM* Continuous Glucose monitoring, *CV* coefficient of variation, *HbA1c* glycated haemoglobin, *HDL* high density-lipoprotein, *IFG* impaired fasting glucose, *IGT* impaired glucose tolerance, *LDL* low density-lipoprotein, *MAGE* mean amplitude of glucose excursion, *MBG* mean blood glucose, *MPMG* mean post-meal maximum glucose, *NGT* normal glucose tolerance, *SD* standard deviation

### Primary endpoint

**Fig. 1:**
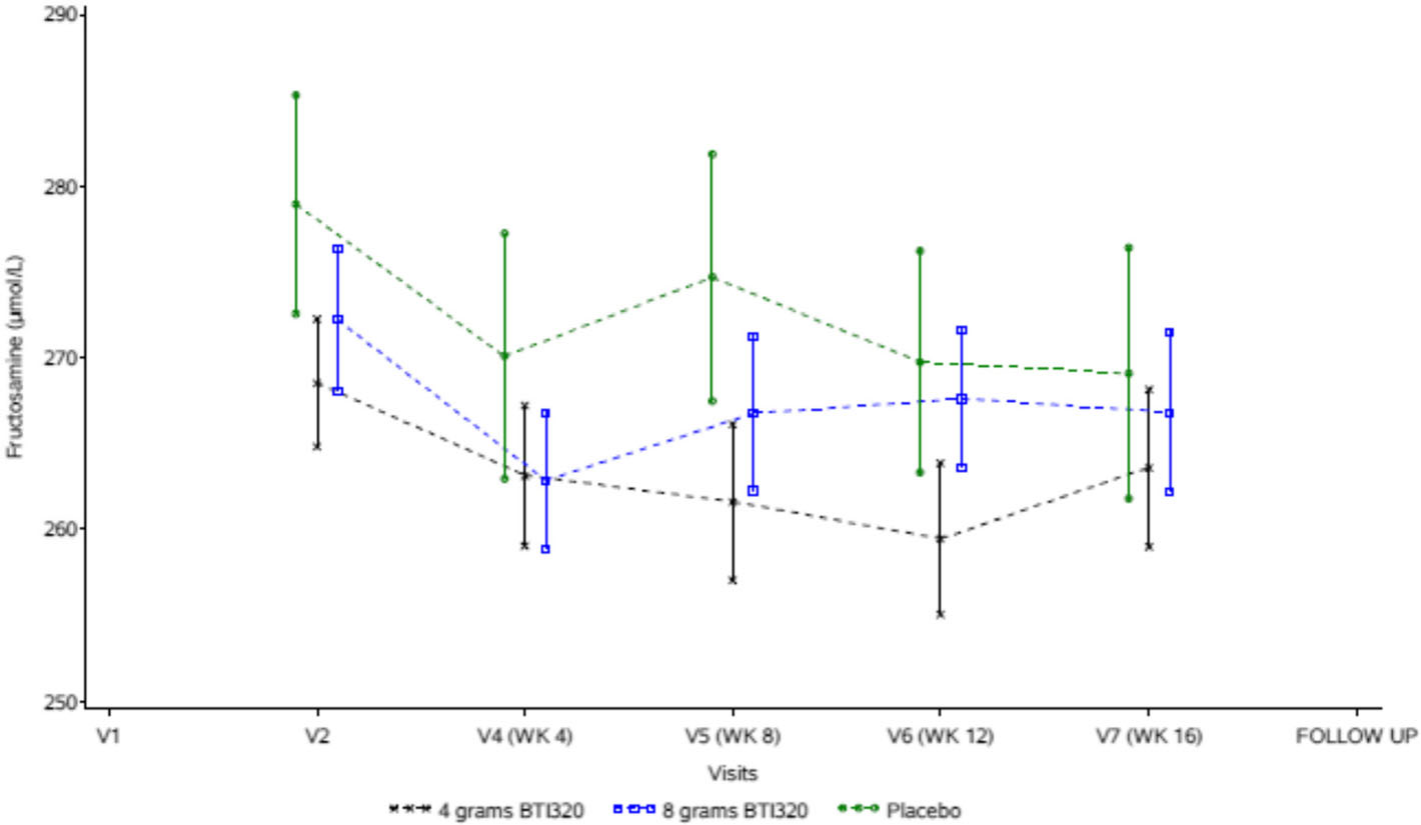
Changes in serum fructosamine levels from baseline in low dose BTI320, high dose BTI320 and placebo groups

In the intention-to-treat analysis, changes in serum fructosamine levels from baseline to 4 weeks were − 5.2, − 9.4 and − 8.8 μmol/L in subjects receiving low dose BTI320, high dose BTI320 and placebo, respectively (Fig. 1). The estimated mean differences in the change in serum fructosamine levels from baseline to 4 weeks were not significant for the comparison between low dose BTI and placebo (mean difference 2.5 [95% confidence interval {CI} -6.3, 11.2] μmol/L, *p* = 0.57) and between high dose BTI and placebo (mean difference − 1.6 [95% CI -10.3, 7.1] μmol/L, *p* = 0.72), adjusted for gender, age, and baseline fructosamine (Table 2, Fig. 1). Analysis of the *per protocol* population yielded similar results.

**Table 2:** Changes in glycemic and metabolic indices from baseline between low dose or high dose BTI320 and placebo in the intention-to-treat analysis

Clinical Variable	Low Dose BTI320				High Dose BTI320			
	Week 4		Week 16		Week 4		Week 16	
	Mean Difference (95% CI)	p*	Mean Difference (95% CI)	p*	Mean Difference (95% CI)	p*	Mean Difference (95% CI)	p*
Serum fructosamine, μmol/L	2.46 (−6.28, 11.20)	0.57	1.14 (−9.17, 11.45)	0.83	−1.57 (− 10.3, 7.11)	0.72	− 0.92 (− 11.1, 9.27)	0.86
HbA1c, %	**–**	**–**	− 0.01 (− 0.13, 0.10)	0.83	**–**	**–**	− 0.04 (− 0.16, 0.08)	0.48
2-h glucose AUC post-MTT, mmol/L×min	65.0 (− 14.4, 144.5)	0.11	−13.6 (− 99.6, 72.3)	0.75	68.9 (− 9.9, 147.7)	0.09	40.0 (−43.9, 123.9)	0.34
2-h insulin AUC post-MTT, mIU/L×min	1219.2 (− 1129.0, 3567.7)	0.30	−980.0 (− 2604.0, 643.8)	0.23	1111.2 (− 1211.0, 3433.2)	0.34	323.9 (− 1269.0, 1916.6)	0.68
2-h C-peptide AUC post-MTT, μg/L×min	128.4 (−22.4, 279.2)	0.09	15.0 (− 99.6, 129.6)	0.79	101.8 (− 47.3, 250.9)	0.18	60.1 (− 52.5, 172.6)	0.29
2-h GLP-1 AUC post-MTT, pmol/L×min	42.8 (− 144.8, 230.3)	0.65	− 120.2 (− 385.3, 144.9)	0.37	32.2 (− 149.7, 214.1)	0.72	49.0 (− 198.1, 296.2)	0.69
Systolic BP, mmHg	−6.4 (− 15.3, 2.5)	0.16	−2.2 (− 9.8, 5.5)	0.57	− 2.4 (− 11.2, 6.4)	0.58	1.0 (− 6.6, 8.6)	0.79
Body weight, kg	0.0 (−1.2, 1.3)	0.95	−1.7 (−3.2, − 0.1)	0.03	0.7 (− 0.5, 1.9)	0.26	− 0.1 (− 1.7, 1.4)	0.86
Total cholesterol, mmol/L	0.06 (− 0.31, 0.43)	0.75	0.03 (− 0.39, 0.44)	0.90	− 0.21 (− 0.58, 0.16)	0.26	− 0.22 (− 0.63, 0.20)	0.30
LDL-cholesterol, mmol/L	0.07 (− 0.28, 0.41)	0.69	0.10 (− 0.28, 0.48)	0.59	− 0.26 (− 0.60, 0.09)	0.14	− 0.18 (− 0.56, 0.20)	0.35
hs-CRP, mg/L	0.77 (−0.79, 2.33)	0.33	0.62 (− 1.32, 2.57)	0.52	0.15 (− 1.39, 1.70)	0.84	1.13 (−0.78, 3.05)	0.24

*The *p*-values of treatment effects were obtained by ANCOVA analysis adjusted for age, gender and baseline measurements

*AUC* area under curve, *BP* blood pressure, *CI* confidence interval, *CRP* C-reactive protein, *GLP-1* glucagon-like peptide-1, *HbA1c* glycated haemoglobin, *hs-CRP* high-sensitivity C-reactive protein, *LDL* low density-lipoprotein, *MTT* meal tolerance test

### Secondary endpoints

Parameters of post-prandial glucose excursion and glycaemic variability were calculated for each subject based on data from CGM. Using ANCOVA with adjustment for gender, age and baseline values, we did not detect significant differences in CGM glycaemic parameters between treatment with low dose or high dose BTI320 and placebo. Using random effect models adjusted for variability by meals, treatment with low dose BTI320 was associated with reduction in 1-h, 2- h and 3-h AUCpp and MPMG compared with placebo by 16 weeks (Table 3). The SDs at 1-h, 2-h and 3-h post-meal were lower in the low dose BTI320 group although the differences just missed statistical significance. Treatment with high dose BTI320 did not differ from placebo with respect to CGM parameters in random effect models.

**Table 3:** Changes in CGM glycaemic indices from baseline between low dose or high dose BTI320 and placebo using random effect models with repeated measurements adjusted for intra-individual between-meal and between meal-day variability

CGM Parameter	Low Dose BTI320		High Dose BTI320	
	Mean Difference (95% CI)	*p*	Mean Difference (95% CI)	*p*
AUCpp at 1 h	−0.30 (− 0.48, − 0.11)	< 0.01	−0.14 (− 0.32, 0.04)	0.13
AUCpp at 2 h	−0.59 (− 1.01, − 0.18)	0.01	−0.17 (− 0.57, 0.24)	0.42
AUCpp at 3 h	−0.74 (− 1.35, − 0.14)	0.02	−0.17 (− 0.75, 0.42)	0.57
MPMG	−0.42 (− 0.81, − 0.03)	0.03	−0.09 (− 0.48, 0.29)	0.63
SD at 1 h	−0.05 (− 0.13, 0.02)	0.18	0.01 (− 0.06, 0.09)	0.73
SD at 2 h	−0.07 (− 0.15, 0.00)	0.06	0.03 (− 0.05, 0.10)	0.46
SD at 3 h	−0.07 (− 0.15, 0.00)	0.06	0.03 (− 0.05, 0.10)	0.46
% CV at 1 h	−0.48 (− 1.46, 0.50)	0.34	0.22 (− 0.73, 1.18)	0.64
% CV at 2 h	−0.62 (− 1.50, 0.26)	0.17	0.39 (− 0.46, 1.24)	0.37
% CV at 3 h	−0.65 (− 1.62, 0.31)	0.18	0.42 (− 0.51, 1.36)	0.37

*AUCpp* post-prandial incremental area-under-curve, *CGM* Continuous Glucose monitoring, *CI* Confidence interval, *CV* coefficient of variation, *MPMG* mean post-meal maximum glucose, *SD* standard deviation

At 16 weeks, serum fructosamine levels were reduced by 5.0 and 6.8 μmol/L in subjects receiving low dose and high dose BTI320, respectively but the changes did not differ from placebo. Similarly, there were no differences in changes in HbA1c from baseline to 16 weeks between intervention with BTI320 and placebo (Table 2). The AUC of glucose, C-peptide, insulin and GLP-1 over 2 h post-MTT were similar in the 3 groups at 4 weeks and 16 weeks (Table 2). At 30 days following treatment completion, 0% of subjects on low dose BTI320, 4.3% of those on high dose BTI320, 0% of those on placebo had normal glucose tolerance and HbA1c < 5.7% (39 mmol/mol).

Body weight was significantly reduced in the low dose but not the high dose BTI320 group. At 16 weeks, the mean change in body weight relative to placebo was − 1.7 (95% CI -3.2, − 0.1) kg in subjects receiving low dose BTI320 (*p* = 0.03) and − 0.1 (95% CI -1.7, 1.4) kg in those receiving high dose BTI320 (*p* = 0.86) (Table 2). There were no differences in changes in total cholesterol, LDL-cholesterol, triglyceride, HDL-cholesterol, urate, hs-CRP, systolic and diastolic BPs between treatment with either doses of BTI320 and placebo. Caloric intake as estimated using food frequency questionnaire as well as self-reported satiety did not differ between the 3 groups.

### Subgroup analysis

Post-hoc subgroup analysis was conducted to test the effects of low dose and high dose BTI320 on changes in 1-h, 2- h and 3-h AUCpp from baseline. Firstly, treatment effects were examined in subjects of high (BMI ≥26 kg/m^2^) and low (BMI < 26 kg/m^2^) BMI (Additional file 2: Table S1). In the high BMI group, BTI320 in both low and high doses reduced AUCpp compared with placebo, whereas in the low BMI group, AUCpp was decreased with low but not high dose BTI320. Next, we examined treatment effects by IFG and IGT status (Additional file 2: Table S2). In the group with IFG and/or IGT, low dose and not high dose BTI320 reduced AUCpp, consistent with results from the main analysis. In contrast, in the group without IFG and IGT, treatment effects were not demonstrated with BTI320 of either doses, although subject number was small in this group (*n* = 18). Lastly, in subgroup analysis conducted in younger (< 59 years) and older (≥59 years) age groups, we detected reduction in AUCpp with high dose but not low dose BTI320 among younger subjects whilst reduction was observed with low dose but not high dose BTI320 in older subjects (Additional file 2: Table S3).

### Safety endpoints

Treatment with BTI320 for 16 weeks had no effects on pre-specified safety parameters of renal function, liver function and blood counts. Significantly more subjects receiving either low or high dose BTI320 reported abdominal distension and increased flatulence (Additional file 2: Table S4). There was no difference in the frequency of gastrointestinal symptoms between the low dose and high dose groups. One subject randomized to low dose BTI320 was diagnosed to have osteosarcoma of the left femur during the study and later required chemotherapy and amputation of the affected leg. The serious adverse event was deemed unrelated to BTI320 as the subject reported history of leg pain at screening prior to commencement of study intervention. Hypoglycaemia was not reported in any of the subjects throughout the treatment period.

## Discussion

In this proof-of-concept study of Chinese subjects with prediabetes, treatment with BTI320 at either low or high doses was not associated with significant changes in serum fructosamine levels compared with placebo at 4 weeks. Despite absence of significant reduction in serum fructosamine, subjects assigned BTI320 experienced less glycaemic variability as evidenced by diminished post-prandial glucose AUC and MPMG during CGM. Post-prandial glucose control is difficult to achieve in individuals with type 2 diabetes and results from the present study suggest that therapeutic action of BTI320 may be extended to this disease population.

### Glycaemic action of BTI320

The glucose-lowering effect of galactomannan in the form of guar gum in patients with type 2 diabetes has been examined in previous studies. In an early double-blind cross-over study of 11 patients with non-insulin treated diabetes, Aro and colleagues observed reduction in fasting and post-prandial glucose following 3 months of dietary supplementation with 21 g of guar gum per day in divided doses [12]. Fuessel and colleagues evaluated the effects of guar gum administered in the form of granules sprinkled over meals at a dose of 5 g per meal in 18 patients with type 2 diabetes and similarly found a diminution of post-prandial glucose AUC when guar gum was consumed prior to standard meal tolerance test [14]. In a single arm study by Groop and colleagues of 15 patients with diet-controlled diabetes, 15 g of guar gum granules per day taken with water or added to food resulted in significant lowering of HbA1c and fructosamine but not fasting plasma glucose over a 48-week intervention period [22]. Dall’alba and colleagues confirmed modest reduction in HbA1c and not fasting glycaemia following 6 weeks’ treatment with 10 g per day of partially hydrolyzed guar gum in a recent study of 44 patients with metabolic syndrome and type 2 diabetes [17]. Although these studies were of small number sample sizes and many were not placebo-controlled, the predominant effect of guar gum supplementation on post-prandial over fasting glucose was consistently observed, in keeping with the proposed interfering action of galactomannan on absorption of carbohydrates in the gastrointestinal tract [10].

In the present study, we detected significant attenuation in several CGM glycaemic variability parameters among subjects with prediabetes receiving low dose BTI320. Accordingly, treatment with low dose BTI320 reduced 1-h, 2-h and 3-h incremental post-prandial glucose AUC by 0.30, 0.59 and 0.74 mmol/L×hour, respectively, compared with placebo. The maximum blood glucose within 3 h post-meal was lowered by 0.42 mmol/L in subjects receiving low dose BTI320 compared with placebo. Reductions were also observed in the high dose group albeit not reaching statistical significance. Contrary to effects on glycaemic variability, there were no significant changes in serum fructosamine or HbA1c at up to 16 weeks of intervention when compared with placebo. The predominant action of BTI320 in suppressing post-prandial glucose excursion might not be of sufficient magnitude to translate into discernable changes in serum fructosamine and HbA1c which comprise both fasting and post-prandial periods of glycaemia. It is also noteworthy that mannans-containing compounds such as BTI320 theoretically blunts post-prandial hyperglycaemia by slowing down the rate of glucose absorption more so than reducing the absolute amount absorbed, which in part explains the absence of reduction in overall glycaemia as measured using conventional glycaemic markers. As we have conducted our study in subjects with prediabetes who have less pronounced glucose fluctuation, this might have limited the study power to demonstrate significant glycaemic effects compared with testing in population with overt diabetes.

In previous investigations of healthy individuals and in patients with type 2 diabetes, addition of guar gum to a standard oral glucose load or test meal dampened post-prandial rise in plasma insulin and gastric inhibitory polypeptide [23, 24]. In our study, the reduction in post-prandial glucose was not accompanied by changes in 2-h AUC of insulin, C-peptide and GLP-1 during MTT, although there were non-significant trends of lower insulin and GLP-1 levels in the low dose BTI320 group compared with placebo.

### Mechanisms of action of galactomannan

The mechanisms of blood glucose lowering and metabolic effects of guar gum have been extensively studied in the last two decades. For instance, galactomannan has been shown to increase viscosity of gastrointestinal content which in turn resists the movement of carbohydrates to the absorptive surface of the intestine, thus reducing accessibility of digestive enzymes to their substrates resulting in delayed glucose absorption [25]. Other evidence suggested that galactomannan may directly bind to and inhibit digestive enzymes such as alpha-amylase [26]. More recently, colonic fermentation of ingested guar gum has been demonstrated to alter short-chain fatty acid composition in the colon and modulate colonic microbiota [27, 28]. For example, consumption of guar gum promotes the production of propionic acid which has been reported to have favorable action on cholesterol and glucose metabolism [29]. Given our increasing knowledge regarding the roles of incretin biology [30] and microbiome [31] on intermediary metabolism as well as the proven effects of alpha-glucosidase inhibitor, a compound sharing similar actions as guar gum, in preventing diabetes in subjects with IGT [7], long-term use of galactomannan has the potential to stall progression to diabetes in at-risk individuals.

### Weight effects of BTI320

Rodents being fed guar gum consistently exhibited reduced food intake and less weight gain [32]. In a small study of 21 obese subjects, administration of 10 g of guar gum twice daily for 8 weeks lowered body weight and was associated with fall in hunger rating [33]. The viscous nature of the food bolus mixed with guar gum slows down gastric emptying which augments satiety and facilitates portion control [34]. In the present study, we also observed modest decrease in body weight in the low dose BTI320 group, although we did not detect differences in caloric intake and in measures of satiety between subjects exposed and those not exposed to BTI320.

### Tolerance

BTI320 was relatively well tolerated. Between 17 and 25% of participants assigned BTI320 developed abdominal distension and 29–33% reported increased flatulence but only 3 subjects had to stop study drug prematurely because of adverse effects. These gastrointestinal symptoms were likely due to increased bacterial digestion of complex carbohydrates in the colon producing gas. Hypoglycaemic symptoms were not reported in any of the subjects. In the STOP-NIDDM study, about one third of participants discontinued acarbose prematurely [7]. Advantages of BTI320 over acarbose which shares similar action mechanisms are the improved tolerability and ease of administration.

### Study limitations

We acknowledge the following limitations of our study. Firstly, as our ultimate goal was to explore the clinical utility of this drug derived from natural compounds in prevention of diabetes, we have only included subjects with prediabetes. As such, our results cannot be extrapolated to people with diabetes. Secondly, fructosamine was used as a measure of short term glycaemia and there are limitations associated with this test. Fructosamine does not fully capture post-prandial hyperglycaemia which may be better reflected using other markers such as 1,5-anhydroglucitol, which were not measured in the present study. Thirdly, only subjects of Chinese ethnicity were tested and our results may not be generalised to people of other ethnic or cultural groups who have different dietary pattern. Fourly, we did not demonstrate a dose-related response and only low dose BTI320 showed statistical efficacy in the reduction of both blood glucose and body weight. The small sample size might have limited the study power to conclusively examine glucose-lowering action of BTI320. Inter-individual variability with respect to meal content, meal size and post-prandial glucose absorption might challenge the strength of the study to assess dose response, particularly if subjects in the three intervention groups might not have been balanced in this respect due to small numbers. Importantly, differences in age, BMI and IFG / IGT status between the three groups at baseline might also have contributed to the unexpected absence of treatment effects with the higher dose in the main analysis. In this regard, post-hoc subgroup analysis was conducted to explore whether treatment effects differ by these parameters. Here, we observed reductions in AUCpp with high dose as well as low dose BTI320 among obese subjects, whilst changes were not seen with high dose BTI320 in the non-obese group, suggesting that baseline BMI is one of the explanatory variables for the lack of treatment effects in the high dose group when the cohort was analysed in its entirety. We speculate that obese subjects, who are likely to have different eating habits to non-obese individuals, derive greater weight and hence glucose benefits than lean subjects.

## Conclusions

In this proof-of-concept study of subjects with prediabetes, low dose BTI320 (4 g three times daily) did not reduce fructosamine levels at 4 weeks as specified in the primary endpoint but attenuated post-prandial rise in blood glucose based on CGM with modest weight loss. Future research will be required to test and confirm the glycaemic and weight effects of BTI320 in a larger sample.

## Additional files

Additional file 1: **Figure S1.** Subject disposition. (DOCX 37 kb)

Additional file 2: **Table S1.** Subgroup analysis (high and low BMI groups) on the changes in post-prandial incremental area-under-curve from baseline between low dose or high dose BTI320 and placebo using random effect models with repeated measurements adjusted for intra-individual between-meal and between meal-day variability. **Table S2.** Subgroup analysis (patients with IFG and IGT, and without with IFG and IGT) on the changes in post-prandial incremental area-under-curve from baseline between low dose or high dose BTI320 and placebo using random effect models with repeated measurements adjusted for intra-individual between-meal and between meal-day variability. **Table S3.** Subgroup analysis (younger and elder groups) on the changes in post-prandial incremental area-under-curve from baseline between low dose or high dose BTI320 and placebo using random effect models with repeated measurements adjusted for intra-individual between-meal and between meal-day variability. **Table S4.** Frequencies of gastrointestinal adverse events among subjects in low dose BTI320, high dose BTI320 and placebo groups. (DOCX 18 kb)

## Abbreviations

ANCOVA: Analysis of Covariance
AUC: Area-under-curve
BP: Blood pressures
CGM: Continuous glucose monitoring
CI: Confidence interval
CUHK: Chinese University of Hong Kong
CV: Coefficient of variations
GLP: Glucagon-like peptide
HbA1c: Glycated haemoglobin
HDL: High density-lipoprotein
hs-CRP: high-sensitivity-C reactive protein
IFG: Impaired fasting glucose
IGT: Impaired glucose tolerance
LDL: Low density-lipoprotein
MAGE: Mean amplitude of glucose excursion
MPMG: Mean post-meal maximum glucose
MTT: Meal tolerance test
OGTT: Oral glucose tolerance test
SD: Standard deviation
