# Supplementary material for: A proof-of-concept study to evaluate the efficacy and safety of BTI320 on post-prandial hyperglycaemia in Chinese subjects with pre-diabetes
Source: BMC Endocr Disord. 2018 Aug 31;18:59. doi: 10.1186/s12902-018-0288-5 (PMC6119318; doi:10.1186/s12902-018-0288-5)
Supplement: Supplementary file 1 — Figure S1. Subject disposition. (DOCX 37 kb) [file 12902_2018_288_MOESM1_ESM.docx]

Figure S1

Subject disposition

Subjects screened

(n=77)

Completed study

(n=12)

Completed study

(n=23)

Completed study

(n=22)

Subjects excluded

(n=17)

Placebo

(n=12)

BTI320 8 gram

(n=24)

BTI320 4 gram

(n=24)

Subjects randomized

(n=60)
