# Supplementary material for: A proof-of-concept study to evaluate the efficacy and safety of BTI320 on post-prandial hyperglycaemia in Chinese subjects with pre-diabetes
Source: BMC Endocr Disord. 2018 Aug 31;18:59. doi: 10.1186/s12902-018-0288-5 (PMC6119318; doi:10.1186/s12902-018-0288-5)
Supplement: Supplementary file 2 — Table S1. Subgroup analysis (high and low BMI groups) on the changes in post-prandial incremental area-under-curve from baseline between low dose or high dose BTI320 and placebo using random effect models with repeated measurements adjusted for intra-individual between-meal and between meal-day variability. Table S2. Subgroup analysis (patients with IFG and IGT, and without with IFG and IGT) on the changes in post-prandial incremental area-under-curve from baseline between low dose or high dose BTI320 and placebo using random effect models with repeated measurements adjusted for intra-individual between-meal and between meal-day variability. Table S3. Subgroup analysis (younger and elder groups) on the changes in post-prandial incremental area-under-curve from baseline between low dose or high dose BTI320 and placebo using random effect models with repeated measurements adjusted for intra-individual between-meal and between meal-day variability. Table S4. Frequencies of gastrointestinal adverse events among subjects in low dose BTI320, high dose BTI320 and placebo groups. (DOCX 18 kb) [file 12902_2018_288_MOESM2_ESM.docx]

Table S1 Subgroup analysis (high and low BMI groups) on the changes in post-prandial incremental area-under-curve from baseline between low dose or high dose BTI320 and placebo using random effect models with repeated measurements adjusted for intra-individual between-meal and between meal-day variability

|  | **BMI < 26kg/m^2^** | | | | **BMI ≥ 26kg/m^2^** | | | |
| --- | --- | --- | --- | --- | --- | --- | --- | --- |
| **CGM Parameter** | **Low Dose BTI320** | | **High Dose BTI320** | | **Low Dose BTI320** | | **High Dose BTI320** | |
|  | **Mean Difference**  **(95% CI)** | **p** | **Mean Difference**  **(95% CI)** | **p** | **Mean Difference**  **(95% CI)** | **p** | **Mean Difference**  **(95% CI)** | **p** |
| AUCpp at 1 hour | -0.37 (-0.64, -0.10) | 0.01 | -0.03 (-0.26, 0.21) | 0.83 | -0.38 (-0.68, -0.08) | 0.01 | -0.33 (-0.64, -0.03) | 0.03 |
| AUCpp at 2 hours | -0.93 (-1.51, -0.35) | <0.01 | -0.11 (-0.41, 0.62) | 0.67 | -0.92 (-1.61, -0.23) | 0.01 | -0.79 (-1.48, -0.09) | 0.03 |
| AUCpp at 3 hours | -1.08 (-1.90, -0.27) | 0.01 | 0.32 (-0.40, 1.04) | 0.38 | -1.46 (-2.48, -0.44) | 0.01 | -1.34 (-2.37, -0.31) | 0.01 |

AUCpp, post-prandial incremental area-under-curve; BMI, body mass index; CGM, continuous glucose monitoring; CI, confidence interval

Table S2 Subgroup analysis (patients with IFG and IGT, and without with IFG and IGT) on the changes in post-prandial incremental area-under-curve from baseline between low dose or high dose BTI320 and placebo using random effect models with repeated measurements adjusted for intra-individual between-meal and between meal-day variability

|  | **IFG and IGT** | | | | **No IFG and IGT** | | | |
| --- | --- | --- | --- | --- | --- | --- | --- | --- |
| **CGM Parameter** | **Low Dose BTI320** | | **High Dose BTI320** | | **Low Dose BTI320** | | **High Dose BTI320** | |
|  | **Mean Difference**  **(95% CI)** | **p** | **Mean Difference**  **(95% CI)** | **p** | **Mean Difference**  **(95% CI)** | **p** | **Mean Difference**  **(95% CI)** | **p** |
| AUCpp at 1 hour | -0.47 (-0.84, -0.09) | 0.02 | -0.06 (-0.41, 0.29) | 0.73 | -0.17 (-0.39, 0.05) | 0.12 | -0.10 (-0.31, 0.11) | 0.35 |
| AUCpp at 2 hours | -1.10 (-1.94, -0.26) | 0.01 | -0.18 (-0.97, 0.60) | 0.64 | -0.23 (-0.70, 0.25) | 0.35 | 0.05 (-0.41, 0.52) | 0.83 |
| AUCpp at 3 hours | -1.28 (-2.49, -0.08) | 0.04 | -0.22 (-1.36, 0.92) | 0.70 | -0.28 (-0.97, 0.41) | 0.43 | 0.16 (-0.52, 0.83) | 0.65 |

AUCpp, post-prandial incremental area-under-curve; BMI, body mass index; CGM, continuous glucose monitoring; CI, confidence interval; IFG, impaired fasting glucose; IGT, impaired glucose tolerance

Table S3 Subgroup analysis (younger and elder groups) on the changes in post-prandial incremental area-under-curve from baseline between low dose or high dose BTI320 and placebo using random effect models with repeated measurements adjusted for intra-individual between-meal and between meal-day variability

|  | **Age < 59 years** | | | | **Age ≥ 59 years** | | | |
| --- | --- | --- | --- | --- | --- | --- | --- | --- |
| **CGM Parameter** | **Low Dose BTI320** | | **High Dose BTI320** | | **Low Dose BTI320** | | **High Dose BTI320** | |
|  | **Mean Difference**  **(95% CI)** | **p** | **Mean Difference**  **(95% CI)** | **p** | **Mean Difference**  **(95% CI)** | **p** | **Mean Difference**  **(95% CI)** | **p** |
| AUCpp at 1 hour | 0.16 (-0.17, 0.48) | 0.34 | 0.19 (-0.15, 0.53) | 0.28 | -0.45 (-0.69, -0.21) | <0.01 | -0.05 (-0.26, 0.17) | 0.67 |
| AUCpp at 2 hours | 0.66 (-0.05, 1.36) | 0.07 | 0.86 (0.12, 1.60) | 0.02 | -0.98 (-1.52, -0.44) | <0.01 | -0.04 (-0.54, 0.45) | 0.86 |
| AUCpp at 3 hours | 0.91 (-0.14, 1.97) | 0.09 | 1.26 (0.15, 2.37) | 0.03 | -1.19 (-1.96, -0.41) | <0.01 | 0.02 (-0.69, 0.74) | 0.95 |

AUCpp, post-prandial incremental area-under-curve; BMI, body mass index; CGM, continuous glucose monitoring; CI, confidence interval

Table 4 Frequencies of gastrointestinal adverse events among subjects in low dose BTI320, high dose BTI320 and placebo groups

| **Adverse Events, % (n)** | **Placebo** | **Low Dose BTI320** | **High Dose BTI320** |
| --- | --- | --- | --- |
| Abdominal distension | 8.3 (1) | 25.0 (6) | 16.7 (4) |
| Abdominal pain | 16.7 (2) | 8.3 (2) | 4.2 (1) |
| Flatulence | 16.7 (2) | 29.2 (7) | 33.3 (8) |
| Frequent bowel movements | 41.7 (5) | 16.7 (4) | 8.3 (2) |
| Diarrhoea | 16.7 (2) | 20.8 (5) | 12.5 (3) |
| Constipation | 8.3 (1) | 4.2 (1) | 0.0 (0) |
